# The New Epoch in Medicine

**Published:** 1889-06

**Authors:** Robert Ormiston


AMERICAN JOURNAL

—OF—

DENTAL SCIENCE.

VOL. XXIII. THIRD SERIES—JUNE, 1889. No. 2.

ARTICLE I.

### THE NEW EPOCH IN MEDICINE.

BY ROBERT ORMISTON, M. D.

[From the Proceedings of the New York Odontological Society, January 15th, 1889.]

A theory is a succinct and comprehensive statement of the underlying principles that pervade phenomena. A theory indicates the mental altitude of the age. It is a focus of the time. It sums up the character of a period. Without a theory, knowledge remains disjointed and chaotic; facts seem unrelated. A theory brings order out of confusion; facts arrange themselves like an army into companies, regiments, and divisions. Everything is put where it belongs, and the whole is a splendid achievement. We know those early Greeks by their theories. We know by their theories how meagre were their facts. They failed not in intellectual grasp, but in *facts*. Even Aristotle, who had collected more facts than any man of his time, could not resist the temptation of constructing the universe in theory before he had laid its foundations in truth. Notwithstanding their failures, we owe these great minds an untold debt of gratitude. We owe to them the methods by which to succeed.

Aristotle, the father of logic, by the triumphant use of the inductive and deductive methods of research bequeathed to posterity the keys of nature. He whispered into the dull ear of the world the "Open Seasame" to the treasures of science. This was the seed-time of our harvest. Every day the world is richer and better because of exact methods of research and reason.

Our own age has formulated four great theories, commensurate with its vast accumulation of facts,—the theory of evolution, the theory of the conservation and correlation of force, the theory of the origin of species, and the germ theory of disease. The germ theory, whatever may be its outcome, is important enough to have produced an epoch in the history of medicine. It has dictated the course of investigation and has revolutionized therapeutics. Surgery within the last fifteen years has become a new art. It has demonstrated the possibility of arresting septic fermentation in wounds, and the capacity of all wounds to heal without suppuration. What are the conditions of septic fermentation? Three,—moisture, warmth, and microbes. Absence of anyone of these conditions is sufficient to prevent decomposition. Under the old regime a surgical wound was a constant invitation to floating germs to set up in business, generating pus, producing the poisonous ptomaines through pathogenic fermentation. Then follow local changes: inflammatory action begins, systemic disturbance known as septic fever sets in, not unfrequently followed by death. The modern treatment of wounds ends all this,—no fever, no formation of pus; a bland and healthy union of the approximate tissues occurs. This treatment is based entirely on the well-known principles which have long been applied to the preservation of organic substances. Aseptic surgery consists in the intelligent use of exsiccation or drying, the actual cautery, and chemical sterilization by germicides; also the combination of chemical sterilization with exsiccation. Modern surgical methods are succinctly expressed in the term *Listerism*. Listerism does not merely mean carbolic acid spray; it means any method of excluding and killing such germs as are liable to set up suppurative fermentation in wounds or in any way hinder reparative action. The aseptic form of treatment is that which preserves a clean wound from septic infection. It is purely preventive. It has been demonstrated that a dustless operating room renders all other precautions unnecessary. Antiseptic treatment is to prevent the further extension of existing trouble. One method prevents the fire, the other extinguishes it. The literature of medicine is filled with triumphal records of septic and antiseptic surgery. The difficult has become easy, that which was dreaded is sought, the impossible is proposed, and the future is all aglow. Who now dreads a compound fracture of the thigh? To-day joints are freely exposed to the atmosphere, offending bacilli vigorously expunged, the cavities filled with an antiseptic solution and closed up with the assurance that there will be no suppurative inflammation.

Perhaps nothing in modern surgery has shown its marvelous progress more than its achievements in laparotomy. It was the *bete noir* of our predecessors It came through the valley and shadow of death to stand at last upon the hill-tops of life. It was not peritonitis that was to be feared; it was microbes and *debris*. A story is told of Mr. Tait, the great laparotomist. Some time ago he was called upon by a celebrated German sugeon, who inquired the secret of his remarkable surgical success. Mr. Tait looked at his German visitor's hands and hesitated at first to answer. Finally he said, "It is because I give the greatest attention to the care of my nails." The German glanced at his hands, and turned away never to return.

Thoroughly cleanse the peritoneal cavity and exclude infection, and a favorable result is almost certain to follow. If one kidney offends and threatens life, the surgeon removes it. If disease obstruct the bowels, he cuts it out and unites the healthy ends, and life is saved. He does not even hesitate to extirpate an unruly spleen. A surgeon can do almost anything he chooses if he conforms to aseptic and antiseptic methods. The steps of a surgical operation are as carefully attended to as if it were a chemical experiment. All the instruments are waiting in trays containing a solution of carbolic acid. The ligatures are boiled in antiseptic fluid, in which they remain until wanted. All bandages, sponges, and dressings are made thoroughly antiseptic. The hands of all those engaged in the operation are disinfected, and from time to time dipped into a carbolic solution. The part to be operated on is carefully washed and shaved; it is surrounded by cloths saturated by some disinfectant solution. The knife of the surgeon is followed by a stream of antiseptic liquid. At the completion of the operation the wound is closed, with every careful detail attended to to make the cut surfaces inaccessible to micro-organisms. This gives you but a faint idea of the scrupulous care necessary. The result is beyond all that the old surgery dreamt of. When one thinks of the surgeon of to-day, it is almost incredible that not more than one hundred and fifty years ago he was found in a barber's shop.

You, gentlemen, belong to the grand army of surgeons. You are engaged in the treatment of lesions more or less surgical. You are in fact medical specialists as much as the oculist, aurist, or orthopedist. I am aware that the intellect of your own department of the healing art is quite abreast of the time in the new thought. But do dentists in every-day practice carry out what they know of aseptic and antiseptic methods? When you think what a forest of bacterial algæ the mouth is, and that many of them are pathogenic, it does not seem possible to lay too much stress on this point. Dr. Sternberg found that in his own saliva there are always enough of what he calls the Micrococcus Pasteuri, when injected into a rabbit, to kill it in forty-eight hours. This particular coccus is now attracting large attention, and may turn out to be the coccus in pneumonia. The condition of the mouth and the character of the bacteria found there will in the near future play a most important *role* in therapeutics. The bacteria of the mouth, besides being pathogenic and benign, are also in a very important degree physiological. We are now anxiously waiting to know just what their functions are in digestion and assimilation. Quite recently Pasteur has isolated as many as seventeen micro-organisms in the mouth which survive the action of the gastric juice; of this number some dissolved albumen, gluten, and casein, and some transformed starch into sugar. From the present indications, the fermentative change they produce in food is a most important feature in digestion. This subject will enlarge the scope of scientific dentistry, and furnish a new field for thought and investigation. We may yet go to the dentist to assist our digestion as well as our trituration..

Let us for a few moments consider these organisms that have of late become so prominent in all medical literature, and which follow us for good or evil from the cradle to the grave. From a morphological point of view they are most insignificant. They are not distinguishable separately by the unaided vision, but in mass they appear quite characteristic in color and arrangement. Their structure is of the lowest kind, having no differentiated organs. They appear to be nothing but very minute filaments of living protaplasm having a delicate covering of cellulose. These minute forms, after much consideration, have been placed in the vetegable kingdom with the genus algæ or seaweed. The various species differ greatly in size, but may be said to average about one-ten-thousandth of an inch in length. Those which are round dots are commonly called cocci. They are very minute, not more than one-twenty-five thousandth of an inch in diameter. Those which have a rod-like form and produce spores are called bacilli, the name bacterium being confined to those species in which the spore formation does not exist. On account of the uncertainty in regard to the existence of spore production in the Schizomycetes, the limit of the genus Bacterium is uncertain, hence it has been proposed to abandon the term altogether and include all the red-forms under Bacilli. The rods when twisted on themselves are called spirilli.

Many bacteria are capable of free movements in fluids, rotating on their longitudinal axes, oscillating forwards and backwards, and conduct themselves as if they possessed organs of motion. That they possess such organs of motion is more than doubtful. What sometimes appear to be such are really thread-like extensions of the soft gelatinous membrane layers. Bacteria multiply with astonishing rapidity,—under favorable circumstances as often as once an hour. The individual cell first elongates until it becomes double its original length; after a short period of repose the shell becomes constricted in the center; at first only the contents but at last the whole cell is divided. Thus it goes on continually. Another method characteristic of bacilli is the formation of spores which form within the cell and escape when the cell-wall is broken up. These spores have a much more pronounced vitality than the cells themselves. They are very difficult to destroy, resisting extremes of temperatures and the action of acids and alkalies.

To the physiologist bacteria are subjects of extreme interest. When one thinks of their astonishing power of changing the whole character of the environment in which they multiply, the occult manner in which they produce the deadly ptomaines, the mysterious character of fermentation which is in numerous instances produced by them, one is profoundly impressed. Such, for example, as the souring of milk or lactic fermentation, the ammoniacal fermentation, the butyric fermentation, the vinous fermentation, the spoiling of meat, fish, and other nitrogeneous substances. In fact, all putrefaction is the result of the ceaseless activity of these countless organisms. When we examine bacteria as pathologists we reach the climax of wonder and dread; we are face to face with the destroyers of the race. Wars, Famine, and nature's most dire cataclysms are nothing when compared with the activites of the pathogenic forms of these organisms. All bacteria are not pathogenic; their great work for good is in the capacity of scavengers. They return the elements of organization back to their original source with renewed activities for fresher and higher combinations. The bacteria have been classified according to their habits in living or dead organisms, so we call them parasites and saprophytes. They are also classed ærobic and anærobic, according as they do or do not require free oxygen for their support. As dentists you are interested in the anærobic saprophytes. To these classes belong the bacteria which produce dental caries.

Here let me say that your *confrere*, Dr. Miller, of Berlin, who I am proud to say is an American, has the honor of establishing the germ theory of dental caries on a sound experimental basis. The dental mind traversed many a sea of theory before it entered the haven of truth. You will all remember the vital or inflammatory theory by Boudet and Jourdain as far back as 1754 to 1766, the chemical theory by Robertson, the *mineral* acid theory of Dr. Watt, of Ohio. Dr. Magitot, of Paris, had an *organic* acid theory produced by fermentation. Monsieur Desirabode opposed all chemical theories on the ground that caries often began in the pulp chamber and therefore could not have been caused by the action of acids. Then the fermentative or putrefactive theory, another kind of chemical theory. After this Liebig's molecular-motion theory, which was spoiled by the discovery of the yeast-plant in 1838 by Schwann; and now we have the parasitic, germ, or septic theory. This entirely accounted for dental carics by the action of micro-organisms, but did not explain how these organisms were able to penetrate the sound enamel. Dr. John Tomes in 1859 advanced a new theory, the chemicovital, which is fully explained in the first edition of his work on dental surgery. As late as 1868 we have the most impossible of all theories, the electro-chemical of Bridgman. The crown of the tooth, according to this theory, becomes the positive electrode, the surrounding tissues the negative; all that is now required to produce dental caries is to have the secretions of the mouth become abnormally acid, and the galvanic processes end in caries. Now we have reached the last, which is termed the chemico-parasitic. The attack to produce dental caries is made by three divisions of micro-organisms: First, those which transform the starches and unfermentable sugar; the second changes the fermentable sugar into lactic acid; the third institutes a digestive fermentation. If the normal secretions of the mouth do not neutralize the effects of the acid generators, in some sequestered spot in the dental array when food has lodged will begin the delicate operation of decalcification of the enamel. Then the door is quietly opened to the dentine, and in rush the waiting micrococci, one-fifth-thousands of an inch in diameter. The dental tubuli are broad avenues to these swarming freebooters. Within the tubuli the process of decalcification continues until no lime-salts are left. The third division finishes the work of destruction, and nothing is left but the pulpy putrescent tooth-cartilage.

Koch has propounded four laws of verification in the investigation of parasitic pathogenic organisms:

"1. The micro-organisms must be found in the blood, lymph, or diseased tissues of the man or animal suffering from or dead of the disease.

"2. The micro-organisms thus obtained must be isolated and cultivated in suitable media,—that is, outside the animal body. These pure cultivations must be carried on through successive generations of the organism.

"3. A pure cultivation thus obtained must, when introduced into the body of the healthy animal, produce the disease in question.

"4. In the inoculated animal the same micro-organism must again be found."

Dr. Miller has triumphantly submitted to the discipline of these rules, as all of you know who have read his published papers. He determined first that the acid-forming ferment in the mouth was capable of self reproduction, and must therefore be of organic origin, and not, as in the case of ptyalin, an unorganized ferment. A second series of experiments demonstrated the fact that must be of great interest to the dentist,—that the organisms producing dental caries do not require oxygen in order to live and thrive. They are just as happy deep down in the bottom of a tooth as in any other place. They go on just as industriously with the lactic acid business as if they were in a more salubrious region. They belong to the anærobic division.

The third series of experiments consisted, first, in producing a pure culture of the bacteria of dental caries by the usual method of bacteriologists. The seed material was taken by a sterilized point from the deepest part of a decaying tooth. A portion of the pure culture obtained was introduced into a tube containing a fermentable mixture; another portion was placed in a similar tube containing a non-fermentable mixture, both being sterilized. In each of these tubes were placed small sections of a sound, recently extracted bicuspid which had been steritized. The first tube became acid in a few hours; the sections of the sound tooth soon softened, and at the end of a week became pliable. At the end of two weeks all but the thicker sections were entirely decalcified and were easily cut with a knife. Sections were made by means of the microtome, which were stained and mounted for microscopic examination. The dental tubules were found to be distended, distorted, and broken down by the ravages of the micro-organisms, resulting in the formation of oval spaces just as you would find them in natural decay. In the second tube, containing the non-fermentable mixture, no change occurred.

Dr. Miller does not pretend to say that parasitic activity supplies all the conditions entering into dental caries. There are of course predisposing causes,—some inborn weakness of structure which, like all weakness, invite attack; an acid condition of the oral secretions, acid food, or medicine may give rise to caries in spots which otherwise might have escaped unharmed. Dr. Miller found micro organisms in every one of a thousand slides of dental caries examined. Another fact which helps to sustain the chemico-parasitic theory is that when a tooth is decalcified by an acid void of fungi the dental tubuli are not changed in diameter, the softened dentine is not discolored, and putrefaction does not occur. To one who has no practical knowledge in your department, but who has gone over the literature of the subject, it would seem conclusive that the parasitic theory has been scientifically demonstrated by Dr. Miller and others who have given experimental attention to the subject.

The more intricate character of constitutional diseases, and the difficulty of producing a general effect profound enough to kill bacteria in the blood without at the same time killing the patient, has no doubt prevented the same sweeping revolution in medical therapeutics that we find in surgery. But even here the practical application of the theory of germs to disease is making progress. Many of the new and incoming medicines are antiseptic and germicidal in intent. Certain forms of indigestion produced by fermentative action are successfully treated by disinfectants. Diphtheria may be arrested by an early and vigorous use of disinfectant measures, and no doubt similar success has been obtained in consumption. It has been found that the best atmosphere for the consumptive is not the high nor the warm nor the cold, but the *aseptic*. I have mentioned that one of the prime conditions for the development of micro-organisms is moisture. For this reason a dry atmosphere becomes antiseptic,—it arrests germ activity. This has led to the very recent method of treating consumption by inhalation of hot dry air. Of course any change, high or low, of the temperature optimum may be enough of itself to destroy the capacity for development of the tubercular bacteria. Dryness alone will do it without regard to temperature. Quite a number of skin-diseases are known to be produced by micro-organisms and cured by germicides. Several diseases of inferior animals have been successfully produced by inoculation by pure culture of bacteria found in these diseases. This is also true of erysipelas, glanders, relapsing fever, and tuberculosis. In the case of typhoid fever, although a constant organism is found, no inferior animal has been discovered susceptible to the typhoid baccillus.

Although I have occupied more of your time than I contemplated when I began this paper, I feel that I have given you a much more imperfect presentation of the subject than I could have wished. It is evident that by the researchess of bacteriologists we are entering a new era in the science and practice of medicine. How great the changes will be that shall come to pass by new investigations it is impossible to forecast. Much has been done, but much more remains to be done. We have not yet determined whether zymotic diseases are produced by the direct action of micro-organisms or by the indirect effect of products produced by their activity of growth and power to decompose the medium in which they flourish. The deadly ptomaines are produced in this way, and products yet unknown may be discovered, so that ultimately the subject may be carried into the domain of organic chemistry.

The President: Gentleman, you have heard this very interesting and delightful paper of Dr. Ormiston's, and the subject is now before you for discussion.

Dr. Perry: It seems to me that in having a scientific man we have almost lost a poet. I do not know whether the spirit of the paper is more sweet and lovely that it is wise; but certainly I am in accord with all that is said in it, with my little knowledge of the subject. I think there are several practical applications of the teachings of the paper which can be made in our specialty; as in the treatment of exposed pulps, in the treatment of the roots of pulpless teeth, and in the care of our dental instruments. Perhaps some of you gentlemen may remember that a number of years ago I ventured to advocate the application of the germ theory in the treatment of roots of pulpless teeth and the troubles that arise therefrom. I mentioned that in my belief the source of troubles of that kind is not to be found in the pulp chamber alone, but also in the substance of the dentine itself. Of course we know that the fashion heretofore has been to direct the entire treatment to the pulp-canal; it was believed that if this was thoroughly cleansed and purified, everything was done that could be. It seems to me that we have not done all that should be expected, because there are innumerable little canals or tubuli opening into the pulp chamber which require and should receive antiseptic treatment also; and if by means of hot air those little tubuli can be dried a little and placed in condition to receive such disinfectants or antiseptic remedies as may be used, it seems to me that that is one good point gained,—if the theory of the paper be correct. I think it is an error to assume that if we cap a pulp once and trouble arises from it, that is all that can be done with it. I have capped the same pulp three or four times, and at last succeeded in saving it; and I believe my final success was due to the fact that at last I had rendered not only the surface of the exposed pulp but the surrounding dentine itself aseptic. I had finally produced such a quiet, undisturbed condition of the tissues that nature reasserted herself and was able to raise the pulp from its feeble condition to a higher and more healthy state. The pulp was kept alive; and I suppose it is admitted that a living pulp is the best filling that a tooth-root can have. And if this theory be true there is no question but that a great gain can be made by thoroughly drying the cavities as a precedent to the use of antiseptic remedies.

There is another point in connection with this theory which it would be well for members of our specialty to remember, and that is that contagious diseases are believed to be propagated by germs. In view of this fact I have standing in a corner of my washstand a large bottle of bichloride of mercury, and my instruments are never used upon different patients, if they have been used upon the tissues of the flesh, until they have been cleansed and dipped in a solution of bichloride of mercury and dried. This care is not always taken with pluggers and other instruments used only upon the dentine and enamel, but my excavators and all instruments that have been used upon the soft tissues are so treated before being used again. I need not say that this is done for the purpose of disinfecting them. I believe in the theory propounded in the paper, and I am not willing to take the risk of the transmission of disease from one patient to another by using in different mouths instruments that have not been cleansed and disinfected. As to the care of the hands and the nails, I think it is impossible to express too strongly the importance of this esthetic feeling of cleanliness.

There is no doubt in my mind that the theory put forth in the paper to-night is true in almost every particular. It seems to me that it must, in the very nature of the case, be true. I would perhaps take a slight exception to the idea suggested in the first part of the paper, that the germ theory is accountable for the decay of teeth. I should say that it was *one* of the factors, and, as the essayist said also a little later, the *principal* factor in that decay. There is not a shadow of doubt in my mind but that Dr. Miller is on the right track, and that we have waited all these years for a final and satisfactory explanation of the cause of the decay of the teeth.

President Howe: Dr. Ormiston's paper has very much of interest in it for us, and certainly the weight of evidence is very largely on the side of a germ cause for almost all the ills that we as dentists know. There has at times, however, seemed to me to be questions arising out of the facts of practice that are difficult to reconcile with this theory. I am quite aware that there may be apparently irreconcilable facts, and a theory still be true; but such facts may as well be kept in mind, to at least suggest that our natural tendency to accept conclusions too eagerly may well be be held in check by the cultivation of judicial caution. If aseptic conditions will prevent dental decay, and copper amalgam is as actively antiseptic as reported by Dr. Miller, then it would seem decay should never occur at the margins of fillings made of this material, but I have seen such decay repeatedly. I hope we will all watch for these points in the use of this material. It has been my practice for years, after removing the decayed dentine from cavities in children's teeth as well as I could,—and that often was very imperfectly,—to pack them with tin foil, especially in the smaller grinding surface cavities, without any attempt to make them dry, or if there was any such attempt it was usually a failure; and yet such fillings in such imperfectly prepared cavities have almost always been efficient for the purpose for which they were made. So the query has come to my mind, and it recurs to-night, as to how this result is to be reconciled with the septic theory of dental decay. And again I recall the fact that iodoform has been a very useful agent for a long time in the dressing of wounds and in its application to the assumed septic conditions of the canals of pulpless teeth. Owing to the good effects of iodoform it has passed for a very active antiseptic agent; but some investigations have shown it to be a very imperfect antiseptic. And yet neither physicians nor surgeons are willing to dispense with it, and dentists who know its value will not abandon its use until they find some other agent that fills its place, which more active antiseptic agents have not yet seemed to do. This raises a query as to the soundness of the antiseptic or Listerian theory, as it is generally accepted. The distinguished surgeon, Lawson Tait, who was quoted by our essayist, evidently rejects part of the

Listerian creed, for it is reported that it is his practice to wash the wound and viscera after his operations in abdominal surgery with tap water,—that is, water drawn from the tap,—without any antiseptic application whatever. He is reported to do this, and to boast of it, as a defiance of Listerism, it would seem.

I suggest these questions not to oppose the theory advanced, nor to call in question the ascertained truths of bacteriology as related to pathology, but to raise a caution signal, lest we drift too easily with the strong current in which we find ourselves. Perhaps Dr. Ormiston can give us some information on these points.

Dr. Ormiston: I do not know that I can give you any information about the value of tin foil. I presume the cavities are cleaned out before the foil is introduced.

President Howe: Sometimes it is rather imperfectly done; and the point is that, as I think, there must be bacteria left there.

Dr. Ormiston: Bacteria do not reach the tubuli until decalcification has occurred in the enamel. Lactic acid fermentation very soon takes place in saccharine or starchy food that may have lodged in the teeth. The way thus being opened, the coccus "a" of Miller enters the dental tubuli and completes the decay.

Dr. Tait does make his boast that he uses tap water in surgical operations; but the basis of all antiseptic treatment is cleanliness, and if you can cleanse the cavity with tap water it is about as well as if you cleanse it with an antiseptic. Of course, there are very few pathogenic organisms after all in the great number of bacteria. Dr. Tait is a very expert operator; he does not take much time, and there is not so much shock to the patients as in many operations performed by others. Shock reduces the tone of the system and allows the entrance of organisms that otherwise would be resisted and excluded by the system. Dr. Tait is very careful in reference to septic precautions; his instruments are very carefully attended to, being immersed in boiled water, his sponges in carbolic lotion, and all the details are looked after. It is necessary to success in such operations to thoroughly cleanse the peritoneal cavity; and water will do it just as well as anything,—*if* you do it. I am quite sure that if he left organisms in the peritoneal cavity he would have trouble.

Dr. Perry: In capping pulps, if we do not thoroughly cleanse the cavity we have trouble. I should be glad to know that iodoform was to be relegated to the shades of oblivion, because of its disagreeable odor. If it is proven to be a weak antiseptic I shall not be sorry; but it would not seem to be so, because of its unquestionable lasting effects. We know very well that if we open a pulp-chamber a long time after it has been treated with iodoform we still get the odor and effects of it, which is not true of carbolic acid and creasote.

I wanted to ask Dr. Ormiston if he would tell us the relative strength or comparative value of antiseptics; which is the best antiseptic now known to the medical and surgical world.

Dr. Ormiston: Bichloride of mercury is by far the most powerful antiseptic that we have; carbolic acid, perhaps, comes next. Carbolic acid, a two or three per cent. solution, is used as an antiseptic wash for the hands of surgeons and for their instruments.

Dr. Perry: What is the usual strength of bichloride that surgeons use?

Dr. Ormiston: One to one thousand, and from that down to one to five thousand, if it is to be used as a wash.

Dr. Perry: Do you use it as strong as one to one thousand as a wash?

Dr. Ormiston: It is used as an application in that strength, especially for the final irrigation of operation wounds, also in abscess cavities.

Dr. Perry: Is it as stable as iodoform would be?

Dr. Ormiston: It is quite stable when kept in alcoholic solution ready for dilution. Sometimes a little salt is added to prevent disintegration.

Dr. C. D. Cook moved a vote of thanks to Dr. Ormiston for his very interesting and excellent paper, which was passed unanimously.—*The Dental Cosmos.*

